# CRISPR-Cas9 interrogation of a putative fetal globin repressor in human erythroid cells

**DOI:** 10.1371/journal.pone.0208237

**Published:** 2019-01-15

**Authors:** Jennifer E. Chung, Wendy Magis, Jonathan Vu, Seok-Jin Heo, Kirmo Wartiovaara, Mark C. Walters, Ryo Kurita, Yukio Nakamura, Dario Boffelli, David I. K. Martin, Jacob E. Corn, Mark A. DeWitt

**Affiliations:** 1 Innovative Genomics Institute, University of California, Berkeley, CA, United States of America; 2 Children’s Hospital Oakland Research Institute, UCSF Benioff Children’s Hospital, Oakland, CA, United States of America; 3 Research Programs Unit, Molecular Neurology and Biomedicum Stem Cell Centre, Faculty of Medicine, University of Helsinki, Helsinki, Finland; 4 Clinical Genetics, HUSLAB, Helsinki University Central Hospital, Helsinki, Finland; 5 Blood and Marrow Transplant Program, Division of Hematology, UCSF Benioff Children’s Hospital, Oakland, CA, United States of America; 6 Cell Engineering Division, RIKEN BioResource Center, Tsukuba, Ibaraki, Japan; 7 Faculty of Medicine, University of Tsukuba, Tsukuba, Ibaraki, Japan; 8 Department of Molecular and Cellular Biology, University of California, Berkeley, CA, United States of America; Southern Illinois University School of Medicine, UNITED STATES

## Abstract

Sickle Cell Disease and ß-thalassemia, which are caused by defective or deficient adult ß-globin (HBB) respectively, are the most common serious genetic blood diseases in the world. Persistent expression of the fetal ß-like globin, also known as 𝛾-globin, can ameliorate both disorders by serving in place of the adult ß-globin as a part of the fetal hemoglobin tetramer (HbF). Here we use CRISPR-Cas9 gene editing to explore a potential 𝛾-globin silencer region upstream of the δ-globin gene identified by comparison of naturally-occurring deletion mutations associated with up-regulated 𝛾-globin. We find that deletion of a 1.7 kb consensus element or select 350 bp sub-regions from bulk populations of cells increases levels of HbF. Screening of individual sgRNAs in one sub-region revealed three single guides that caused increases in 𝛾-globin expression. Deletion of the 1.7 kb region in HUDEP-2 clonal sublines, and in colonies derived from CD34+ hematopoietic stem/progenitor cells (HSPCs), does not cause significant up-regulation of 𝛾-globin. These data suggest that the 1.7 kb region is not an autonomous 𝛾-globin silencer, and thus by itself is not a suitable therapeutic target for gene editing treatment of ß-hemoglobinopathies.

## Introduction

The ß-hemoglobinopathies Sickle Cell Disease (SCD) and ß-thalassemia are genetic blood diseases characterized by defective or deficient adult ß-globin (*HBB*). SCD is a monogenic recessive disorder caused by a single-nucleotide mutation in *HBB*. SCD affects at least 90,000 predominantly African-American individuals in the US and millions more worldwide, predominantly in Africa and Southern India [1–3]. Even in the developed world individuals with SCD experience a greatly reduced quality of life, and suffer an ~30-year decrement in lifespan [4]. ß-thalassemia is characterized by reduced or absent expression of HBB. The disease affects hundreds of thousands worldwide, with approximately 23,000 births annually in its most severe form, transfusion-dependent ß-thalassemia [5]. The disease is often managed with chronic blood transfusions, which eventually leads to iron overload requiring chelation therapy. Currently the only cure for both SCD and ß-thalassemia is allogeneic stem cell transplantation [6].

Both SCD and transfusion-dependent ß-thalassemia can be ameliorated by increased expression of 𝛾-globin. 𝛾-globin is a ß-like globin that, along with α-globin, comprises heterotetrametric fetal hemoglobin (HbF) [7]. HbF inhibits polymerization of sickle hemoglobin (HbS), and levels of HbF above ~20% are associated with a virtual absence of manifestations of SCD and ß-thalassemia. Increased HbF in adults is a positive predictor of survival in patients with SCD [4,8]. In ß-thalassemia, expression of 𝛾-globincan replace the function of deficient ß-globin. Thus, methods to elevate expression 𝛾-globinare of considerable therapeutic interest.

CRISPR-Cas9 is a gene editing technology that enables efficient and facile genome manipulation via targeted generation of a double strand break (DSB) [9,10]. The break is repaired through either of two general pathways: error-prone non-homologous end joining (NHEJ), or accurate homology-directed repair (HDR) [11,12]. Targeting is directed by a 20 nucleotide guide RNA (gRNA) bound by Cas9 and a Protospacer Adjacent Motif (PAM) within the genome [10]. The development of this modular, user-friendly targeting system has led to an explosion of interest in gene editing, including for the treatment of the hemoglobinopathies.

Strategies to drive -globin expression in erythroid cells via *ex vivo* gene editing of hematopoietic stem/progenitor cells (HSPCs) have recently emerged [13]. Decreased expression of *BCL11A*, which encodes transcription factor that is necessary for silencing of 𝛾-globin expression in adult-stage erythrocytes [7,14], leads to high-level expression of 𝛾-globin. Other efforts have targeted the ß-like globin locus, reproducing naturally occurring HPFH mutations in the promoters of the two 𝛾-globin genes, either through precise genome editing [15,16], or through NHEJ-mediated indel formation to disrupt transcription factor binding sites entirely [17–19].

We used gene editing to explore a potential approach to create an HPFH phenotype. We compared naturally-occurring deletions causing either HPFH or ß-thalassemia identified a candidate 1.7 kb intergenic repressor region upstream of the *HBD* gene [20] (Figure a in S1 File). We found that CRISPR-Cas9 deletion of this region and specific sub-regions induced expression of HbF in heterogeneous pools of HUDEP-2 cells. However, multiple clonal HUDEP-2 sublines harboring a deletion of the 1.7 kb region did not exhibit increased HbF. We also observed little up-regulation of 𝛾-globin expression when the deletions were made in CD34+ hematopoietic stem and progenitor cells (HSPCs), after differentiation into erythroid colonies and erythroblasts. These results suggest that this 1.7 kb region may contribute to developmental silencing of 𝛾-globin but is not an autonomous 𝛾-globin silencer.

## Results

### Defining a minimal *HBG*-*HBD* intergenic region associated with 𝛾-globin silencing

**Fig 1:**
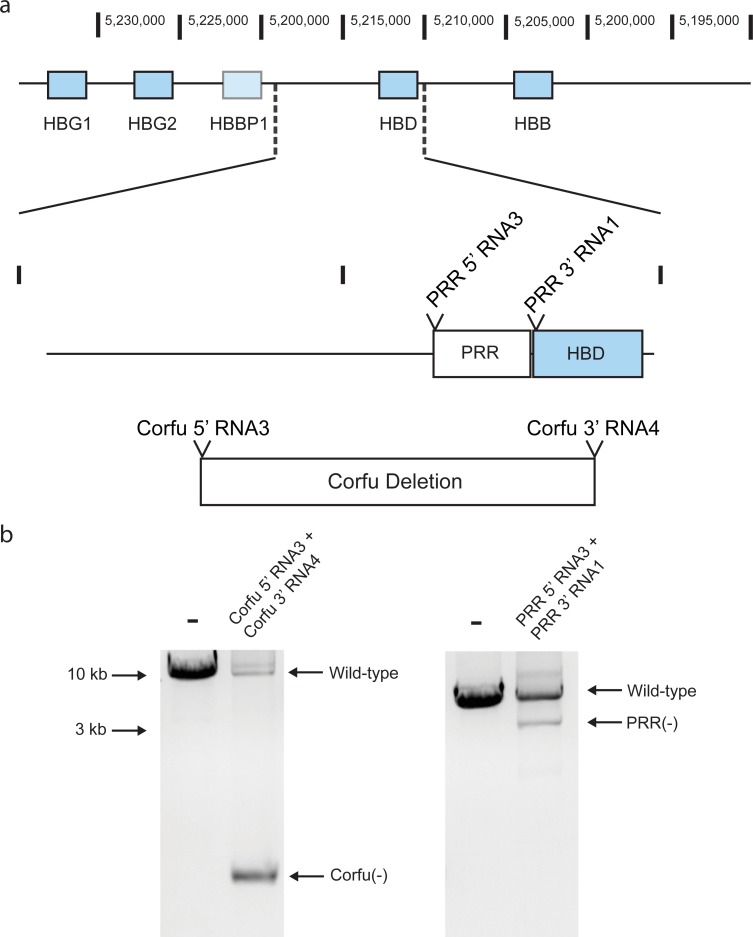
Targeted deletion of genomic regions implicated in 𝛾-globin silencing using selection-free Cas9 RNP electroporation. A) Schematic depicting two targeted regions: the naturally-occurring Corfu HPFH deletion and the 1.7 kb putative repressor region (PRR) upstream of HBD, with the 2 pairs of cuts attempted shown *(13)*. B) PCR amplification after gene editing with pairs of Cas9 RNPs that delete the Corfu and PRR regions with primers outside the targeted regions. Deletion is indicated by the presence of bands with greater mobility corresponding to the deleted alleles which give rise to shorter PCR products.

We began by examining breakpoints of naturally-occurring HPFH deletions to define a minimal region upstream of *HBD* whose deletion is associated with increased 𝛾-globin expression. Individuals lacking the intergenic region between the δ-globin (*HBD*) and 𝛾-globin (*HBG1* and *HBG2*) genes exhibit an HPFH phenotype, in particular the 7.2 kb “Corfu” deletion [21] and several more extended deletions including HPFH-1 and the French HPFH deletion [18,22]. In contrast, deletions that do not include this region are not associated with high HbF expression and instead cause ß-thalassemia [20]. Both HPFH deletion mutations and ß-thalassemia deletions include ß-globin. We hypothesized that these two effects, induction of 𝛾-globin and deletion of ß-globin, were spatially distinct and separable: deletion of regions associated with HPFH can independently up-regulate 𝛾-globin even in the presence of an intact ß-globin gene. We used the HbVar database [23] to identify an exact 1.72 kb genomic region between the 5’ breakpoint of the French HPFH deletion and the Macedonian δ/ß-thalassemia deletion (Figure a in S1 File). This region also appeared to harbor a binding site for Bcl11a in a ChIP-Seq analysis [20]. Hereon we refer to this 1.7 kb region as the Putative Repressor Region, or “PRR” (Fig 1A). The small size of the PRR makes it attractive for genetic dissection, unlike larger deletions [18,19,24]. We sought to explore the relationship between PRR deletion genotypes and globin expression phenotypes.

### Up-regulation of fetal hemoglobin in heterogeneous pools of edited HUDEP-2 cells

As a model system to assess 𝛾-globin expression we used the recently-developed HUDEP-2 cell line, which can be differentiated into late-stage erythroblasts [25]. These cells display an adult pattern of globin expression, with ß-globin expressed and 𝛾-globin silenced. HUDEP-2s can express 𝛾-globin after various genetic manipulations in *cis* or *trans* to the ß-globin locus, and have been used to explore genotype-phenotype relationships related to globin switching [17,26,27]. To edit HUDEP-2 cells, we used Cas9 RNP electroporation, which we have found to be effective at gene targeting in cell lines and CD34+ HSPCs [28–30]. Our goal was to genetically dissect the PRR to identify small regions whose deletion would activate 𝛾-globin, and by extension HbF, expression. We designed Cas9 RNPs and Cas9 RNP pairs to target progressively smaller regions, starting with the full PRR, moving to overlapping sub-regions of the PRR, and culminating in individual Cas9 RNP electroporation of a single sub-region.

**Fig 2:**
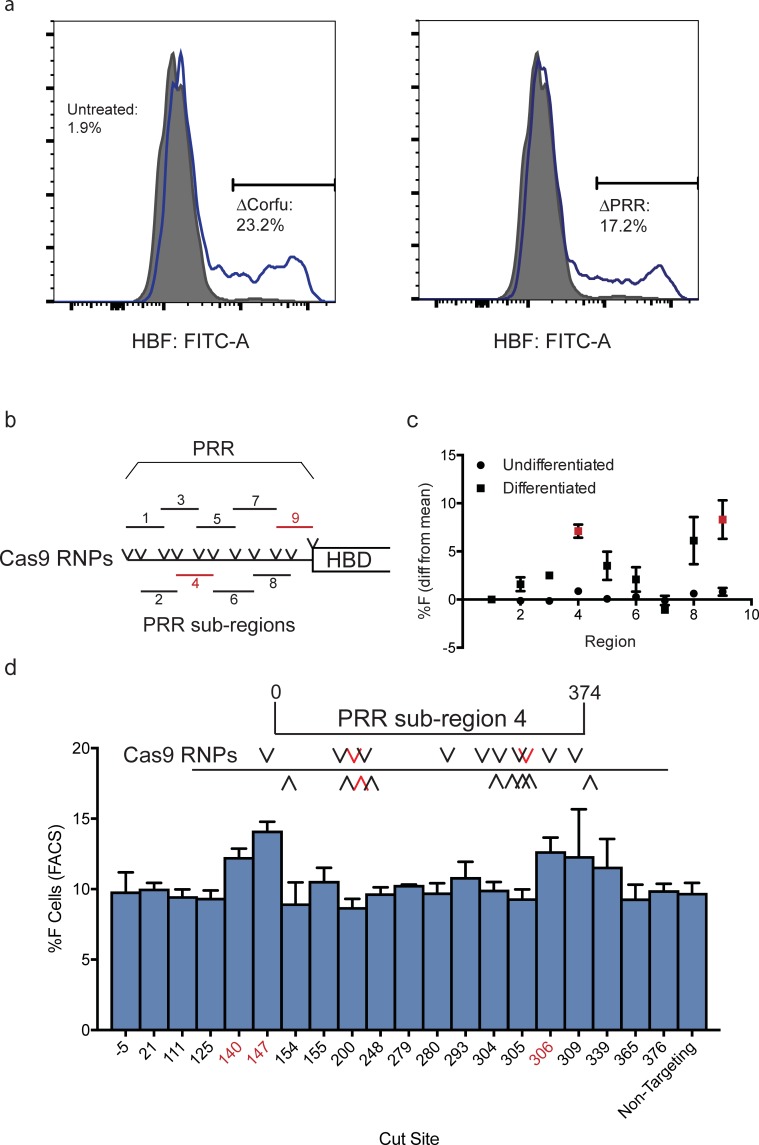
Interrogation of the PRR in the parent HUDEP-2 cell line. A) Representative intracellular FACS plots showing a population of HbF-expressing HUDEP-2 cells, after electroporation of RNP pairs generating each deletion and differentiation into erythrocytes. B) Schematic depicting the PRR, divided into 9 overlapping sub-regions. Deletion of each sub-region is programmed by a pair of RNPs. Sub-region deletions leading to statistically significant increase in HbF expression are marked in red. C) Flow cytometry enumeration of HbF-expressing HUDEP-2 cells after introduction of Cas9 RNPs driving deletion of each sub-region, before and after differentiation into erythroblasts. Results are (mean of each culture)-(mean of all cultures) ± s.d. for 3 biological replicates, regions 4 and 9 (in red) deletion led to a statistically significant increase in HbF expressing cells, compared to the mean of all cultures. D) Scanning mutagenesis screen using 20 separate Cas9 RNPs targeting every PAM in region 4 to differentiated HUDEP-2 cells, with HbF-expressing cells enumerated by flow cytometry. Each RNP is denoted by its cut site within the PRR region 4, from -4 (4 nt upstream of the 5’ region breakpoint) to 376 (376 nt downstream of the 5’ region breakpoint). To ensure consistency, synthetic RNAs were used. Results are mean ± s.d. for 3 biological replicates. Statistically significant sites are marked in red.

We generated Cas9 RNP pairs that cut at the 5’ and 3’ ends of the PRR, and the naturally occurring Corfu deletion (Fig 1B and Figure b in S1 File, guides in Table c in S1 File [21]). Electroporation with pairs of RNPs in this manner can lead to deletion of the intervening sequence, and has been used to reproduce naturally-occurring mutations in earlier studies [18]. Efficient editing by individual candidate guide RNAs was assayed with T7 endonuclease I (T7E1) digest, and guides with >50% editing at each end were paired (Figure b in S1 File). Deletion of the PRR or Corfu region in cell pools was confirmed by the presence of a shorter DNA fragment on an agarose gel following PCR amplification of the targeted regions (Fig 1C). Pools of HUDEP-2 cells electroporated with these pairs of deletion-forming Cas9 RNPs were differentiated into erythrocytes to assess HbF expression by intracellular flow cytometry with an HbF-specific antibody. The edited cell pools displayed an increased proportion of cells expressing HbF (Fig 2A, and Figure b in S1 File) [31]. 17.2% of cells expressed HbF when the PRR deletion RNPs were delivered, and 23% of cells expressed HbF when the Corfu deletion RNPs were delivered, compared to 1.9% of cells for untreated cells.

We further interrogated the PRR by dividing it into 9 overlapping sub-regions and targeting each sub-region separately. In an attempt to enhance deletion, we designed 150 nucleotide single strand oligonucleotide donors (ssODNs) for HDR. These ssODNs had one half matching 75 bp upstream of one breakpoint, and the other half matching 75 bp downstream of the other breakpoint, and thus corresponding to the sequence of a deleted allele (Fig 2B, Table c in S1 File). It is possible that these ssODNs may increase the likelihood that the dual DSBs generated by two RNPs are resolved in favor of a precise deletion, although this hypothesis was not tested in this study (Fig 2B). We evaluated guides by T7E1 digest of genomic DNA. Cultures of HUDEP-2 cells were treated with each pair and the appropriate stapler ssODN and then differentiated. Of each RNP/ssODN set, those programming deletion of regions 4 and 9 were associated with a significant increase in the frequency of HbF-positive cells by flow cytometry, increasing expression by 7% above the mean (of all cultures) for region 4, and 6.5% for region 9 (Fig 2C). RNPs that delete regions that overlap regions 4 and 9 also led to an increase in HbF-expressing cell frequency. Based on these observations, we focused our subsequent studies on region 4, which was the sub-region associated with the largest increase in fetal hemoglobin expression. Note that these screening studies did not definitively establish a link between deletion of any region and an increase in HbF expression.

Deletion of genomic elements using pairs of Cas9 RNPs is useful for defining regions of phenotypic interest. However, two DSBs in one targeted cell could be resolved to a variety of genotypes besides simple deletion: inversions, duplications, and short insertions or deletions at either cut site [19,32]. To avoid these complications, a single cut site is preferred. Hence we attempted to identify a single gRNA within the PRR sub-region 4 that could induce a significant increase in HbF expression. We synthesized 20 Cas9 RNPs to target every wild-type Cas9 PAM within sub-region 4 (Fig 2D and Table c in S1 File). We introduced each of these RNPs individually to HUDEP-2 cells by electroporation in biological triplicate. Gene editing by these RNPs was confirmed by T7 endonuclease I digest (Figure c in S1 File). All but two RNPs (cutting at -5 and 21 bp from the 5’ breakpoint of region 4) led to indel formation within sub-region 4. and analyzed HbF using intracellular flow cytometry. After differentiation into erythrocytes, we found that three RNPs yielded modest but statistically significant increases in the frequency of HbF-expressing cells, when compared to a non-targeting control RNP (Fig 2D).

### Gene edited HUDEP-2 clonal cell lines lacking the PRR or two of its subregions do not consistently up-regulate HbF

We next sought to relate loss of one or more regions within the PRR to 𝛾-globin (and by extension HbF) expression. Our results suggested that the full PRR and discrete sites within it may be involved in silencing of 𝛾-globin in HUDEP-2 cells. However, a given RNP pair can generate many alleles: translocations, inversions, or indel mutations at either cut site. Results from gene-edited polyclonal pools cannot distinguish between these, and so do not clearly show whether a region is required for 𝛾-globin silencing. Indeed, we found evidence of short indel (but not inversion) genotypes when we used amplicon next generation sequencing (NGS) to genotype edited cell clones isolated from these pools (Table b in S1 File), but did not find evidence of long deletions, as has been reported elsewhere [33]. We therefore examined detailed genotype-phenotype relationships through generation and evaluation of edited HUDEP-2 derived clonal cell lines targeting either the PRR or sub-regions 4 or 9.

HUDEP-2 cells are a polyclonal pool of erythroblasts immortalized by lentiviral transduction [25]. We first subcloned HUDEP-2, and selected one clone that retained differentiation and hemoglobinization capabilities, henceforth termed “H2.1”. This and the parental HUDEP-2 cell line were used in subsequent studies.

**Fig 3:**
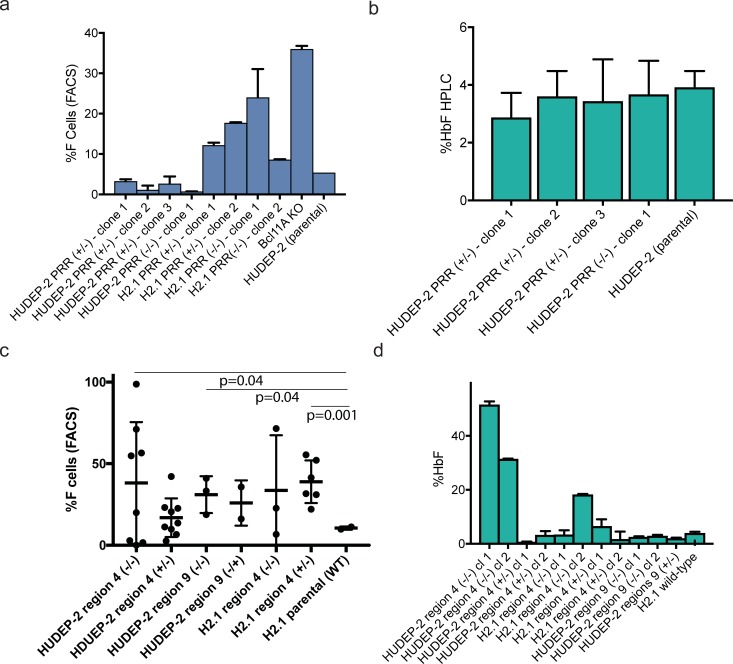
Interrogation of HUDEP-2 and H2.1 cell clones carrying deletions of the PRR and selected sub-regions. A) Proportion of HbF-expressing cells assayed by flow cytometry in cell clones carrying the indicated PRR deletions (mean ± s.d. of 3 biological replicates). B) Quantitation of HbF expression by HPLC in selected clones carrying the indicated PRR deletions, (mean ± s.d. of 3 technical replicates). No significant differences in HbF expression were seen in comparison to parent HUDEP-2 cells. C) Expression of HbF in clonal cell lines carrying the indicated sub-region deletions, measured by flow cytometry, error bars represent mean ± s.d. of 2–8 independently-derived clones for each genotype. Clones with statistically significant differences in HbF-expressing cells compared to unedited H2.1 cells are noted (unpaired t-test). D) Expression of HbF in selected clonal cell lines carrying the indicated sub-region deletions, measured by hemoglobin HPLC Each bar represents the mean ± s.d. of 3 technical replicates for each clone. Phenotypic information for each clone is in Table a in S1 File.

We delivered PRR deletion RNP pairs to a HUDEP-2 pool and to H2.1, isolated edited clones by limiting dilution, and genotyped the resulting clonal lines by PCR amplification and multiplexed next-generation amplicon sequencing (Table a in S1 File). We obtained multiple heterozygous and homozygous deletion clones derived from both HUDEP-2 pool and and the H2.1 subclone (Table a in S1 File). We differentiated several sets of these clones into erythroblasts and assessed globin expression by flow cytometry for HbF-expressing cells and HPLC for hemoglobin expression. While homozygous deletion of the PRR increased HbF expression in a flow cytometry assay in some of the clones, other clones showed no increase in HbF expression (Fig 3A). By HPLC assay, deletion of the PRR in select clones derived from HUDEP-2 failed to induce HbF at all (Fig 3B). This is in stark contrast to the results we obtained from deleting the same region in cell pools. These results suggest that deletion of the PRR alone is not sufficient for 𝛾-globin de-repression in HUDEP-2 cells.

We also generated clonal cells with deletions of the two candidate sub-regions of the PRR (4 and 9) identified by screening in the parent HUDEP-2 line (Figure d in S1 File), editing both HUDEP-2 (sub-region 4 and 9) and H2.1 (for sub-region 4 only). Deletions were confirmed by next-generation sequencing of a PCR amplicon encompassing the full PRR (Table a in S1 File, and supplementary material). We obtained 8 homozygous deletion and 9 heterozygous deletion clones of region 4 in HUDEP-2, 2 homozygous and 7 heterozygous deletion clones of region 4 in H2.1, and 3 homozygous and 2 heterozygous deletion clones of region 9 in HUDEP-2. We note that even in most “homozygous” clonal cell lines, two distinct genotypes are often seen, as the deleted regions vary by 1–10 bp (Table a in S1 File). In all 32 of the 37 clonal cell lines have two distinct genotypes, suggesting these lines are accurately genotyped (HUDEP-2 cells are diploid). We saw no evidence of any unexpected large deletions in any cell line within the 2.5 kb region analyzed by next-generation sequencing [33]. After derivation, clonal cell lines were differentiated into erythroblasts, and HbF-expressing cells were counted by flow cytometry (Fig 3C). Some clones homozygous for sub-region 4 deletion exhibited increased proportion of HbF-expressing cells (with up to 40% for cells derived from HUDEP-2, but not for lines from H2.1, Fig 3C). However, other deletion clones did not exhibit any apparent increase in HbF. Overall, we observed large variation in HbF-expressing cells between clonal cell lines with the same genotype, whether derived from the polyclonal HUDEP-2 line or the isogenic H2.1 sub-clone.

We used HPLC to measure globin content in erythroid cell differentiated from edited HUDEP-2 and H.2 clones (Fig 3D, traces in Figure e in S1 File). Two HUDEP-2 derived clones with homozygous knockouts in sub-region 4 exhibited variable proportions of HbF (1.67% to 98.7%, average 41.8%). But heterozygous sub-region 4 deletion clones showed no increase in HbF, nor did any H2.1-derived clones with homozygous knockouts in sub-region 4. Our results highlight significant clonal differences in HbF expression between sub-clones of HUDEP-2 cells, since H.21 is itself a sub-clone of HUDEP-2. Taken together, our data also suggest that sub-regions 4 and 9 of the PRR are not autonomous silencers of 𝛾-globin silencing in HUDEP-2 cells, since homozygous deletions of these regions leads to increased HbF expression in cell pools but variable expression between edited clonal cell lines.

#### Deletion of PRR or two PRR sub-regions in erythroid colonies derived from CD34+ HSPC does not up-regulate HbF

**Fig 4:**
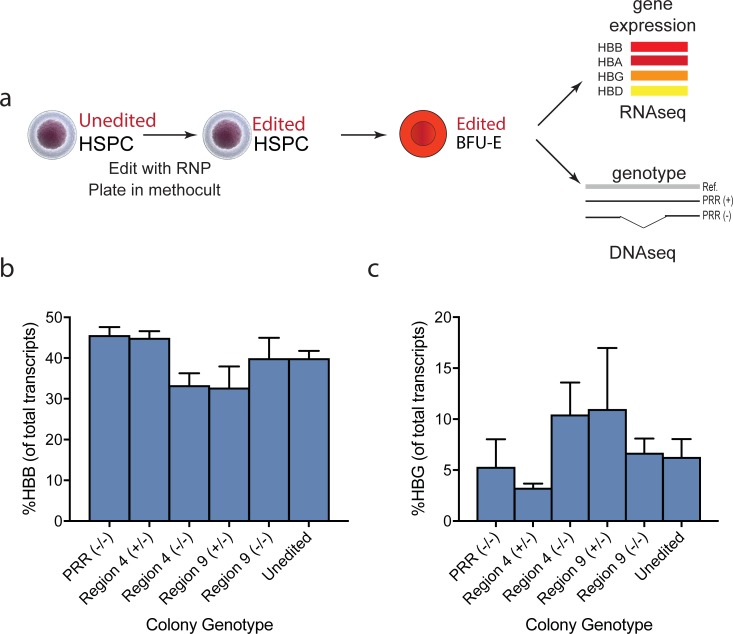
Genotype-phenotype relationships in clonal BFU-E colonies derived from edited hematopoietic stem/progenitor cells. A) Workflow depicting erythroid colony (BFU-E) formation genotype/phenotype assay using edited HSPC. HSPC were edited using RNPs targeting the desired region, plated in methocult, and colonies were grown for 14 days. Erythroid colonies were picked and genotyped by next-generation sequencing and phenotyped for their globin gene expression profile using RNA-seq. B) ß-globin (HBB) expression in erythroid colonies after gene editing, determined by RNA-seq. There was no statistically-significant difference in expression between each genotype of colony. C) 𝛾-globin (HBG1+HBG2) expression in erythroid colonies after gene editing, determined by RNA-seq. Because of the high variation between colonies of the same genotype, there was no statistically-significant difference in expression between each genotype of colony. To control for effects of RNP electroporation, the unedited cells were electroporated with a non-targeting RNP against BFP.

Finally, we assessed whether some PRR or PRR sub-region deletion genotypes can alter 𝛾-globin expression in erythroid colonies derived from human primary CD34+ HSPCs. We edited adult human CD34+ HSPCs with Cas9 RNPs pairs targeting either the PRR, sub-region 4 or sub-region 9 [28] and plated the cells sparsely in methocellulose to form clonal colonies (CFUs). We picked erythroid colonies (both BFU-E and CFU-E), genotyped each colony using PCR and amplicon NGS, and determined globin gene expression using RNA-seq (Fig 4A) [18]. We used pairs of Cas9 RNPs to target deletion of three regions: the full 1.7 kb PRR, sub-region 4, and sub-region 9. ß-globin (*HBB*) expression ranged from 23% to 50% of globin transcripts but was not significantly altered between unedited cells and PRR homozygous knockouts, sub-region four heterozygous or homozygous knockouts, and sub-region nine heterozygous or homozygous knockouts. (Fig 4B and Table b in S1 File). γ-globin (HBG1+HBG2) expression was variable between clonal erythroid colonies and ranged from 0.5% to 26% of globin transcripts (Fig 4C and Table b in S1 File). There was no clear association between PRR deletion genotype and 𝛾-globin expression: homozygous deletion of sub-region 9 did not affect 𝛾-globin expression, while heterozygous deletion may have, and vice versa for deletion of sub-region 4. No deletion genotype led to a statistically-significant increase in γ-globin expression compared to unedited colonies. Any increase in γ-globin expression is obscured by the high variation between colonies, and does not reach therapeutic levels. To confirm whether variation between clones masked the effects of PRR deletion, we differentiated unselected pools of CD34+ HSPC in erythroblasts following editing with either PRR-editing RNP pairs or a non-targeting control guide against BFP (Figure f in S1 File). 𝛾-globin mRNA ranged from 1.5% of globin transcripts on average (control) to 2.8% (PRR deletion). These results suggest that neither the full PRR nor either of the selected sub-regions are in and of themselves necessary for stable 𝛾-globin silencing in primary human erythroblasts.

## Discussion

Here we explored a potential approach to activate expression of 𝛾-globin in adult erythroid cells via targeted ablation of a 1.7 kb potential repressor region (PRR) upstream of *HBD*. We identified the PRR from analysis of naturally occurring human genotypes that lead to HPFH (Figure a in S1 File) [20]. In bulk populations of the HUDEP-2 polyclonal cell line we found that deletion of the PRR and smaller sub-regions led to 𝛾-globin re-expression. However, we found that edited HUDEP-2 clones or edited erythroid colonies derived from CD34+ HSPCs were not consistently associated with increased 𝛾-globin expression. Instead, we found marked heterogeneity in 𝛾-globin expression between clones (or clonal colonies) of the same genotype (and ultimately isolated from the same individual), including among wild-type sub-clones and clonal colonies. We conclude that deletion of the PRR alone is not sufficient for up-regulation of 𝛾-globin to therapeutic levels.

The possibility remains that the PRR cooperates with other regions within or downstream of HBB to mediate 𝛾-globin silencing. Previous studies have shown that deletions including both the PRR and all or part of ß-globin gene, along with other downstream elements, can induce 𝛾-globin [18,19]. In this scenario, 𝛾-globin expression is dependent on *both* ß-globin loss and loss of the PRR. However the efficient induction of more extended deletions encompassing both the PRR and the ß-globin gene may be more challenging in a clinical setting.

The region containing the PRR was implicated in 𝛾-globin silencing on the basis of two lines of evidence. One was the comparison of multiple large deletions in the ß-globin locus: large deletions that do not include the PRR cause ß-thalassemia, while other deletions that do include this region confer a more benign HPFH phenotype [18,20,22]. The second was a ChIP-chip analysis that identified binding of Bcl11a within the PRR [20]. Bcl11a is well documented as a regulator of developmental 𝛾-globin silencing [34], and so this result seemed consistent with the idea that the region was required for developmental silencing. However a more recent study that used an orthogonal method to map Bcl11a binding sites did not identify a binding within the PRR [35].

The broad variation in HbF expression we observed between wildtype HUDEP-2 derived subclones and among wild-type erythroid colonies in HSPC suggests that 𝛾-globin expression level is prone to large spontaneous changes even in cell lines or colonies derived from cells with the same genotype. This suggests that caution should be used in interpreting selected results from HUDEP-2 subclones or HSPC colonies. It is not clear why this is the case, nor why there was a trend indicating that functional elements resided sub-regions 4 and 9 and their overlapping neighbors. Dual guide editing using Cas9 has the potential to generate complex genotypes, including inversions, although no inversions were seen in our NGS analysis [19,32]; it is possible that a minority of these events had an outsize effect in edited pools of cells. A recent study suggested that large deletions and other rearrangements may occur following editing with a single guide, although we do not see direct evidence for this in our NGS analysis, which utilized a 2.5 kb PCR amplicon [33]. Our investigation of clonal cell lines and clonal colonies focused on frank deletion genotypes, and it is possible that more complex genotypes led to 𝛾-globin re-expression in subsets within edited pools of cells, although we saw no evidence of these genotypes in our NGS data. Regardless, our study suggests that the PRR is not an autonomous 𝛾-globin silencer.

While this manuscript was in preparation a new study exploring deletions of larger genomic regions encompassing the PRR was reported using similar methods [19]. Interestingly, deletion of the 7.2 kb Corfu region or a 3.5 kb region that included the 1.7 kb PRR did not lead to significant fetal hemoglobin re-expression in HUDEP-2 cells. Instead a much larger 13.2 kb deletion that encompassed the adult ß-globin promotor did lead to robust 𝛾-globin expression. However, this large deletion also led to 3-fold drop in ß-globin expression, similar to the HPFH-1 deletion using CRISPR/Cas9 in HSPCs [18]. These results and others suggest that simultaneous deletion of the PRR and disruption of ß-globin expression may synergize to lead to high levels of 𝛾-globin re-expression.

## Materials and methods

### HUDEP-2 Cells and cell culture

HUDEP-2 cells were cultured and differentiated according to published protocols [25], with the exception that doxycycline was included in *both* differentiation and expansion media. Expansion medium: 1 μM dexamethasone, 1 μg/mL doxycycline, 50 ng/mL stem cell factor (Peprotech, Inc.), 5 U/mL EPO (Amgen, pharmaceutical grade), in SFEM medium (Stem Cell Technologies). Differentiation medium: 5% human serum (Sigma Aldrich), 2 IU/mL heparin (Sigma Aldrich), 10 μg/mL Insulin (Sigma Aldrich), 5 U/mL EPO (Amgen), 500 μg/mL holo-transferrin (Sigma Aldrich), 1 μM mifepristone (Sigma Aldrich), 1 μg/mL doxycycline in IMDM media (with Glutamax, Gibco, Inc.). Differentiation medium was prepared with one week of use and sterile-filtered using a 0.22 μm filter. To differentiate HUDEP-2 and H2.1 cells from expansion culture, cells were pelleted (300 x g, 5 minutes), thoroughly decanted, re-suspended in differentiation medium at a density of <1,000,000 cells/mL, and cultured for 5 days before analysis for hemoglobin expression by flow cytometry or HPLC.

### Synthesis of Cas9 RNPs

Cas9 RNP component synthesis and assembly was carried out based on published work, and are available online at https://www.protocols.io/groups/igi/protocols [28,36]. Cas9 was prepared by the UC Berkeley Macro Lab using a published protocol [36]. Cas9 was stored and diluted in sterile-filtered Cas9 Buffer (20 mM HEPES pH 7.5, 150 mM KCl, 1 mM MgCl_2_, 10% glycerol, 1 mM TCEP). sgRNA was synthesized by assembly PCR and *in vitro*-transcription. A T7 RNA polymerase substrate template was assembled by PCR from a variable 59 nt primer containing T7 promotor, variable sgRNA guide sequence, and the first 15 nt of the non-variable region of the sgRNA (T7FwdVar primers, 10 nM, Table c in S1 File), and an 83 nt primer containing the reverse complement of the invariant region of the sgRNA (T7RevLong, 10 nM), along with amplification primers (T7FwdAmp, T7RevAmp, 200 nM each). Phusion high-fidelity DNA polymerase was used for assembly (New England Biolabs, Inc.). Assembled template was used without purification as a substrate for *in vitro* transcription by T7 RNA polymerase using the HiScribe T7 High Yield RNA Synthesis kit (New England Biolabs, Inc.). Resulting transcriptions reactions were treated with DNAse I, and purified either by with a 5X volume of homemade SPRI beads (comparable to Beckman-Coulter AMPure beads), and eluted in DEPC-treated water. sgRNA concentration was determined by fluorescence using the Qubit RNA BR assay kit (Life Technologies, Inc). Cas9 RNP was assembled immediately prior to electroporation of target cells (see below). To electroporate a 20μL cell suspension (see below) with Cas9 RNP, a 3.75 μL solution containing a 1.2–1.3X molar excess of sgRNA in Cas9 buffer was prepared. A 3.75 μL solution containing 75 pmol purified Cas9 in Cas9 buffer was prepared and added to the sgRNA solution slowly over ~30 seconds, and incubated at room temperature for >5 minutes prior to mixing with target cells. For electroporation with pairs of Cas9 RNPs, half the amounts and volumes above were used to assemble RNPs, which were then mixed after assembly. For studies depicted in Fig 2E (saturating mutagenesis of PRR sub-region 4), RNA was generously provided by Synthego, Inc. For electroporation of HSPCs (Fig 4), synthetic sgRNA was purchased from Synthego, Inc. Synthesis of RNPs from synthetic sgRNA was accomplished in an analogous manner, using stocks of dried RNA hydrated to 45 μM in water or 0.5X TE.

### Electroporation of HUDEP-2 Cells with Cas9 RNP using the Lonza 4d electroporator

HUDEP-2 cells were cultured in expansion medium prior to editing to mid-log phase (200,000–1 million cells per mL of culture). To electroporate, 100,000–200,000 cells were spun at 300xg for 5 minutes, and resuspended in 20 μL of Lonza P3 solution and 7.5 μL of Cas9 RNP solution prepared as described above, before transfer of the entire 27.5 μL mixture to a small-scale Lonza S electroporation cuvette. Cells were electroporated using Lonza 4d code DD100. Cells were cultured a minimum of 2 days before differentiation and phenotyping analysis.

### Generation of clonal HUDEP-2 sublines

Gene-edited HUDEP-2 and H2.1 cells were cloned by limiting dilution into 96 well plates. After ~2 weeks the cultures were passaged and half of the cells were removed for DNA extraction and PCR (see above). Genotyping was initially performed with standard PCR and agarose gel electrophoresis using primers for the indicated deletion (Table c in S1 File). Clones with desired genotypes by PCR were confirmed with next-generation sequencing (see below).

### Assay of HbF-positive HUDEP-2 cells by intracellular flow cytometry

FACS for HbF was adapted from existing protocols [26]. Differentiated or undifferentiated HUDEP-2 cells were pelleted by centrifugation at 500 x g for 5 minutes, washed with PBS/0.1% BSA, pelleted again and resuspended in 200 μL 0.05% glutaraldehyde (freshly prepared from evacuated ampules of 20% glutaraldehyde from Sigma Aldrich) in PBS and incubated for 10 minutes to fix the cells. Cells were pelleted at 600 x g for 5 minutes and resuspended in 200 μL of 0.1% Triton X-100 in PBS/0.1% BSA and incubated for 10 minutes to permeabilize cells, and pelleted at 600 x g once again before resuspension in 50 μL of anti-fetal hemoglobin-FITC antibody at a 1–10 dilution in PBS/0.1% BSA (BD Biosciences, Inc. clone 2D12) and incubated for 20 minutes to stain cells. After staining, cells were washed three times in PBS/0.1% BSA before analysis by flow cytometry.

### Quantification of relative hemoglobin expression with HPLC

HPLC analysis for hemoglobin expression in differentiated cells was run essentially as described in previous work [37]. Hemolysates for HPLC were prepared from 1–5 million differentiated cells pelleted and resuspended at 100,000 cells/μL in hemolysate reagent (Helena Laboratories), incubated for 10 minutes at room temperature, and clarified by centrifugation. 10 μL of lysate was applied to the HPLC column for hemoglobin analysis. For HPLC a PolyCAT A column 3.54 (PolyLC, Inc.) was used with mobile phase A for loading (20 mM Bis-tris, 2 mM NaCN, pH6.8) and B for elution (20 mM Bis-tris, 2 mM NaCN, 200 mM NaCl, pH 6.9), flow-rate 1.5 mL/min, and detection of hemoglobin by absorbance at 415 nm, and gradient from 0% to 100% mobile phase B over 30 minutes to separate hemoglobins. Hemoglobins were identified based on mobility using a separate FASC hemoglobin standard (Trinity biotech).

### Electroporation of CD34+ HSPCs with Cas9 RNPs

HSPC electroporation was as described [28]. CD34+ HSPCs (G-CSF mobilized, Allcells, Inc.) were thawed and cultured in SFEM (StemCell Technologies, Inc.) with CC110 cytokines (Stem Cell Technologies) for 2 days at a density of <500,000 cells/mL before editing. 100,000–200,000 HSPCs were re-suspended in 20 μL of P3 solution and 7.5 μL of Cas9 RNP or RNP pairs assembled as described above. Because ssDNA reduces viability when gene editing HSPCs, no “stapler” ssDNAs were used to edit HSPCs. For HSPCs, only synthetic sgRNA was used (Synthego, Inc.). HSPCs in P3 solution with RNP were electroporated using code ER100 on a Lonza 4d electroporator. After electroporation, cells were layered with 75 μL of SFEM with CC110 and incubated for 5 minutes at room temperature before transfer to culture in SFEM with CC110. Cells were cultured for 1 day before plating on MethoCult Express at 250 cells/well in a 6 well plate. A subset of the culture (~50,000 cells) was taken to confirm deletions by PCR. After 14 days, individual BFU-E were picked and dispersed in 100 μL of PBS. Half of the suspension was taken for genotyping by genomic PCR and next-generation sequencing, and the other half was stored at -80. Colonies found to have the desired genotypes by genomic DNA PCR were thawed and prepared for RNA-seq, and had their genotypes confirmed by next-generation sequencing before completion of RNA-seq data analysis (see below).

### RNA-seq of erythroid colonies

Total RNA was isolated from individual BFU-E with the ArrayPure Nano-scale RNA purification kit (Epicentre), and converted into cDNA following the Smart-seq2 protocol [38]. The cDNA was fragmented (median size of 170 bp) and ligated into Illumina sequencing libraries with the KAPA HyperPlus kit (KAPA Biosystems). Libraries were sequenced on an Illumina HiSeq4000 apparatus to produce single-ended reads of 50 nucleotides. Reads were aligned with kallisto version 0.43.1 [39] against a reference sequence consisting of the main isoforms of each globin gene at the α- and ß-globin loci. The following Ensembl transcript IDs were used: ENST00000320868.9 (HBA1), ENST00000251595.10 (HBA2), ENST00000199708.2 (HBQ1), ENST00000356815.3 (HBM), ENST00000354915.3 (HBZP1), ENST00000252951.2 (HBZ), ENST00000335295.4 (HBB), ENST00000380299.3 (HDB), ENST00000454892.1 (HBBP1), ENST00000330597.3 (HBG1), ENST00000336906.4 (HBG2), ENST00000292896.2 (HBE1).

Globin transcripts have high levels of sequence identity; to evaluate the ability of kallisto to correctly assign a read to the transcript from which it was derived, we carried out simulations using computationally generated 50-base reads. The globin transcripts listed above were mixed in known proportions that simulate relative transcript levels in erythrocytes (HBA-1: 0.7, HBA-2: 0.7, HBM: 0.01, HBB: 1.00, HBD: 0.04). HBG-1 and HBG-2 were varied between 0.005 and 0.10 to simulate the effect of varying levels of 𝛾-globin expression in a mixture. Sequence segments of 50 nucleotides in length were sampled randomly from this mixture and from its reverse complement (number of samples = 1,000,000), and aligned as described above. The random sampling procedure was repeated 10 times for each mixture. For each alignment result, we calculated the mean ratio of observed over expected counts for each transcript, which we used as adjustment factors for the counts reported by kallisto after aligning real data; the difference in transcript counts before and after adjustment ranged from -1% to 0.5%. The computed adjustment factors were: HBA-1: 1.0397665108; HBA-2: 0.8890515518; HBM: 0.8195218055; HBB: 1.0401825121; HBD: 1.1146242591; HBG-1: 1.321849175; HBG-2: 1.0079414563

### T7 endonuclease assay

To select optimal gRNAs, genomic cutting using Cas9 RNPs was estimated using T7 endonuclease I assay. Genomic DNA was extracted from edited cells (HUDEP-2 or CD34+ HSPC) using QuickExtract solution (Epicentre, Inc.) to a density of >2,500 cells/μL. A PCR amplicon of the targeted region was generated using GXL Primestar Polymerase (Takara, Inc.), 30 PCR cycles, and manufacturer’s instructions, at a final volume of 50 μL using 5 μL of extracted genomic DNA as template. 7 μL of unpurified PCR was mixed with water and 1X NEB buffer 2 to a final volume of 10 μL before re-annealing in a thermal cycler (95 degrees for 5 minutes, cool to room temperature over 10 minutes). 1 μL of T7 endonuclease I was added (NEB, Inc.) and reaction was digested for 30 minutes at 37°C before visualization on a 2% agarose gel or 4–20% polyacrylamide TBE gel (Life Technologies, Inc.). Cutting was compared to PCR amplicons from untreated cells.

### Genotyping of HSPC colonies and HUDEP-2 clonal cell lines edited at the PRR and sub-regions by PCR amplicon deep sequencing (NGS)

Genomic DNA preparation and PCR were performed identically as for T7 endonuclease digest above, except that the PRR primers were used for all samples (see supplemental text for unedited amplicon sequence). The 2.3 kb amplicon was purified, fragmented to an average length of ~350 bp using a Covaris instrument, and prepared for NGS using the Illumina TruSeq Nano HT kit. Libraries (1 for each colony or clonal cell line) were sequenced on an Illumina MiSeq (2x300 paired-end sequencing). Demultiplexed and adaptor-trimmed reads were aligned to the reference amplicon sequence, and the genotype determined by visual inspection of aligned reads in IGV viewer software.

## Supporting information

S1 File Figures a-f Tables a-c, and Supplemental Information.
(DOCX)
