# Supplementary material for: CRISPR-Cas9 interrogation of a putative fetal globin repressor in human erythroid cells
Source: PLoS One. 2019 Jan 15;14(1):e0208237. doi: 10.1371/journal.pone.0208237 (PMC6333401; doi:10.1371/journal.pone.0208237)
Supplement: S1 File — Figures a-f Tables a-c, and Supplemental Information. (DOCX) [file pone.0208237.s001.docx]

**Supporting information**


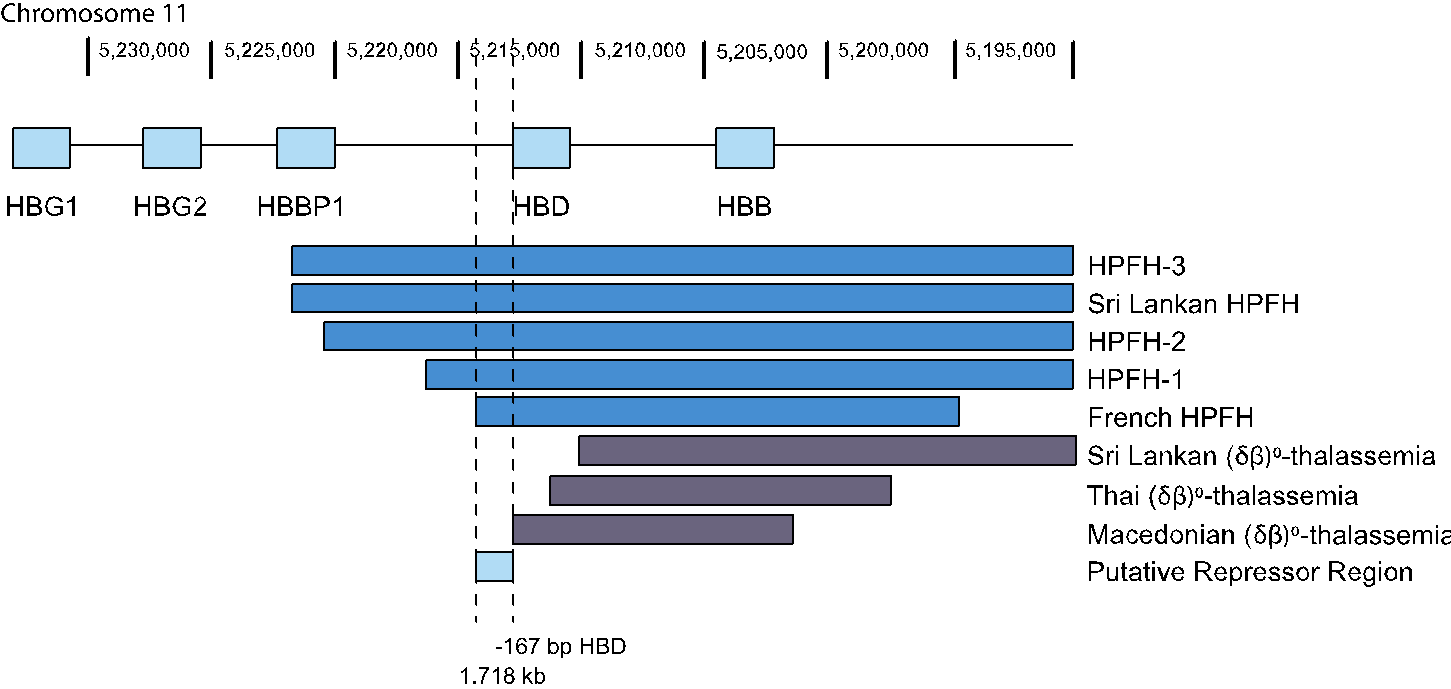


**Fig a.** **Defining a putative repressor region (PRR) from naturally-occurring deletion HPFH mutations**. Figure is based upon *(13)* deletions found in the HbVar database. Mapping of observed mutations associated with ß-thalassemia (grey bars) and observed mutations associated with HPFH (blue bars), it appears that deletion of a 1.72 kb region starting 167 bp upstream of the HBD gene is associated with an HPFH phenotype, while deletions that retain this region are associated with a ß-thalassemia phenotype.


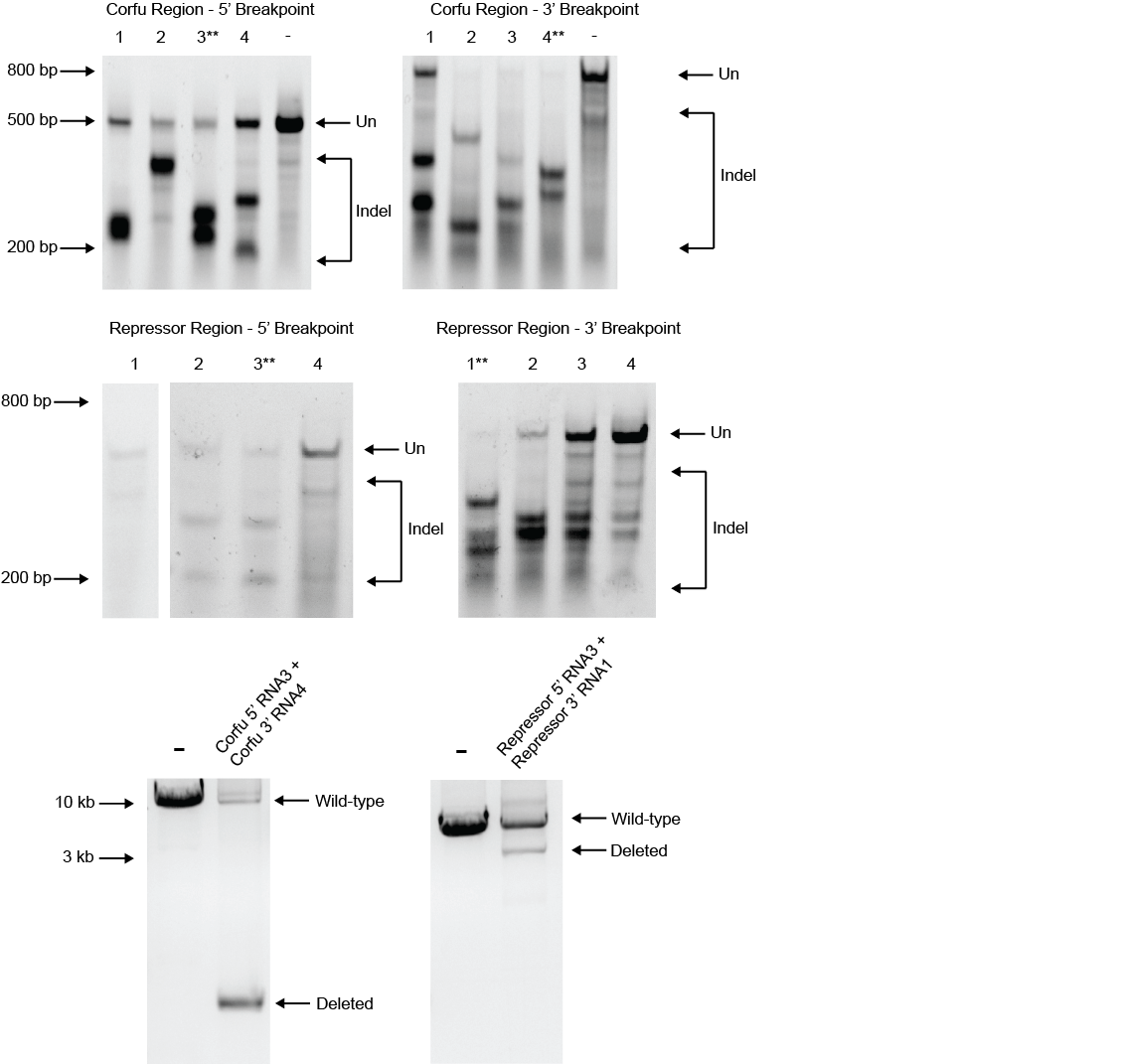


**Fig b. Development of RNP pairs deleting two HPFH-associated regions**. Guides targeting the 4.5 kb Corfu deletion were electroporated in RNPs and assessed by T7 endonuclease digestion of PCR amplicons. Guides with the most efficient targeting as measured by T7 digestion were used as pairs to target the full regions. For the Corfu region, guide 3 on the 5’ side and guide 4 were selected (top row). For the PRR, guide 3 on the 5’ end (1.8 kb upstream of HBD) and guide 1 on the 3’ (adjacent to HBD) were selected (middle row).

**Fig c. T7 endonuclease digest of genomic DNA from HUDEP-2 cells electroporated with Cas9 RNPs targeting region 4 of the PRR, related to Figure 2D**. Cut sites are as indicated in Figure 2D and in the text. Sites where targeting led to statistically-significant increase in HbF expression are highlighted in red.

**Fig d. Development of RNP pairs deleting sub-regions within the putative repressor region (Related to Fig. 3)**. 4 guides targeting each breakpoint were synthesized by *in vitro* transcription, formed into RNPs and delivered to subcloned HUDEP-2 cells by electroporation. 48 hours after electroporation, cells were harvested and the targeted regions were amplified by genomic PCR and edited in individuals RNPs was assessed by T7 endonuclease I digest (above). Optimized paired RNPs were co-delivered by electroporation and deletion was observed with genomic PCR (below).

**Fig e. Representative of HPLC traces of HUDEP-2 clones with the full PRR or either of sub-regions 4 or 9 deleted, as indicated**. Hemoglobins were identified with FASC standards run separately.

**Fig f. RNA-seq of cultures of CD34+ HSPC after electroporation with PRR deletion RNPs, compared to control cultures electroporated with non-targeting (BFP) RNP.** CD34+ HSPC were electroporated with the indicated RNPs, and the entire culture was differentiated into erythroblasts using standard techniques, with no selection for edited cells. Fetal globin (HBG1/HBG2) expression is very low for all cultures, and does not approach any reasonable threshold for therapeutic relevance.

**Table a. NGS genotyping results of clonal cell lines derived in this study**. NGS genotypes are Insertions (“I”) or deletions (“D”) of the indicated nucleotides at the indicated positions within the repressor region NGS amplicon (see below).

| **Clonal cell line** | **NGS genotype** | **NGS allele 1** | **NGS Allele 2** | **%F cells (FACS)** | **%HbF (HPLC) mean ± s.d.** |
| --- | --- | --- | --- | --- | --- |
| HUDEP-2 region4 (-/-) cl 3 | Region 4 (-/-) | D661:1033 |  | 0.0 |  |
| HUDEP-2 region4 (-/-) cl 4 | Region 4 (-/-) | D661:1036 | D666:1040 | 54.8 |  |
| HUDEP-2 region4 (-/-) cl 5 | Region 4 (-/-) | D663:1048 | D667:1037 | 2.9 |  |
| HUDEP-2 region4 (-/-) cl 6 | Region 4 (-/-) | D666:1036 | D663:1040 | 20.0 |  |
| HUDEP-2 region4 (-/-) cl 1 | Region 4 (-/-) | D664:1038, I(CT):661, I(GG):1039 | D667:1038 | 71.1 | 51.53 ± 1.23 |
| HUDEP-2 region4 (-/-) cl 7 | Region 4 (-/-) | D663:1047 | D667:1138 | 56.6 |  |
| HUDEP-2 region4 (-/-) cl 2 | Region 4 (-/-) | D667:1038, D665:665 | D665:1036, I(CT):661 | 98.7 | 31.42 ± 0.18 |
| HUDEP-2 region4 (-/-) cl 8 | Region 4 (-/-) | D650:1043 | D669:1040, D667 | 1.6 |  |
| HUDEP-2 region4 (+/-) cl 1 | Region 4 (+/-) | D673:1040 | I(G):667, I(A):1040 | 23.1 | 0.67 ± 0.14 |
| HUDEP-2 region4 (+/-) cl 3 | Region 4 (+/-) | D662:1071 | D663:669 | 2.6 |  |
| HUDEP-2 region4 (+/-) cl 4 | Region 4 (+/-) | D665, D667:1038 | D659:668, D1038:1040 | 11.2 |  |
| HUDEP-2 region4 (+/-) cl 2 | Region 4 (+/-) | D667:1038 | D664:666 | 42.1 | 3.21 ± 1.54 |
| HUDEP-2 region4 (+/-) cl 5 | Region 4 (+/-) | D665, D667:1038 | D667:669 | 6.6 |  |
| HUDEP-2 region4 (+/-) cl 6 | Region 4 (+/-) | D664:668, D785:1041 | D667, D813-819 | 22.6 |  |
| HUDEP-2 region4 (+/-) cl 7 | Region 4 (+/-) | D666:1038 | D659:668, D1039:1041 | 20.5 |  |
| HUDEP-2 region4 (+/-) cl 8 | Region 4 (+/-) | D666:1038 | D666:670, D1012:1052 | 13.6 |  |
| HUDEP-2 region4 (+/-) cl 9 | Region 4 (+/-) | D667:1038 | D664:666 | 9.8 |  |
| H2.1 region 9 (-/-) cl 2 | Region 9 (-/-) | D1502:1844 | WT | 33.3 | 2.90 ± 0.41 |
| H2.1 region 9 (-/-) cl 1 | Region 9 (-/-) | D1511:1844 | WT | 41.0 | 2.47 ± 0.32 |
| H2.1 region 9 (-/-) cl 3 | Region 9 (-/-) | D1511:1844 | WT | 18.8 |  |
| H2.1 region 9 (+/-) cl 1 | Region 9 (+/-) | D1511:1844 | WT | 16.1 |  |
| H2.1 region 9 (+/-) cl 2 | Region 9 (+/-) | D1286:1646 | WT | 35.7 | 1.98 ± 0.30 |
| H2.1, wild-type | ND | ND | ND | 14.3 | 3.91 ± 0.57 |
| HUDEP-2 PRR (+/-) - cl 1 | Repressor (+/-) | D178:1961 | D1951:1961 | 3.3 | 3.80 ± 0.74 |
| HUDEP-2 PRR (+/-) - cl 2 | ND | ND | ND | 1.1 | 2.76 ± 0.81 |
| HUDEP-2 PRR (-/-) - cl 1 | Repressor (-/-) | D178:1961 |  | 0.7 | 3.97 ± 0.90 |
| HUDEP-2 PRR (+/-) - cl 3 | Repressor (+/-) | D178:1961 | WT | 2.6 | 3.67 ± 1.33 |
| H2.1 PRR (+/-) - cl 1 | ND | ND | ND | 12.2 |  |
| H2.1 PRR (+/-) - cl 2 | ND | ND | ND | 17.7 |  |
| H2.1 PRR (-/-) - cl 1 | ND | ND | ND | 24.0 |  |
| H2.1 PRR (-/-) - cl 2 | ND | ND | ND | 8.6 |  |
| H2.1 Bcl11A KO | ND | ND | ND | 36.0 |  |
| HUDEP-2 (parental), wild-type | ND | ND | ND | 5.4 |  |
| H2.1 region 4 (-/-) cl 1 | Region 4 (-/-) | D666:1038 | WT | 22.7 | 3.39 ± 1.65 |
| H2.1 region 4 (-/-) cl 2 | Region 4 (-/-) | D650:1041 | D666:1038 | 71.5 | 18.22 ± 0.26 |
| H2.1 region 4 (+/-) cl 1 | Region 4 (+/-) | D666:1038 | D663:669 | 52.1 | 6.49 ± 2.61 |
| H2.1 region 4 (+/-) cl 2 | Region 4 (+/-) | D650:1041 | D659:668, D1028:1048 | 55.4 | 1.66 ± 2.88 |
| H2.1region 4 (-/-) cl 3 | Region 4 (-/-) | D666:1038 | D658:665, D702:1038 | 6.7 |  |
| H2.1 region 4 (+/-) cl 3 | Region 4 (+/-) | D666:1038 | D666:669, D1038 | 31.6 |  |
| H2.1 region 4 (+/-) cl 4 | Region 4 (+/-) | D666:1038 | D661:681 | 41.4 |  |
| H2.1 region 4 (+/-) cl 5 | Region 4 (+/-) | D661:1040 | D657:669, D1031:1041 | 22.1 |  |
| H2.1 region 4 (+/-) cl 6 | Region 4 (+/-) | D646:1119 | D662:683, D1016:1053 | 31.0 |  |

**Table b. NGS genotyping results of clonal erythroid colonies (CFU-E and BFU-E) derived from HSPCs after editing with Cas9 RNP pairs programming deletion of the three regions and sub-regions of interest in this study**. NGS genotypes are Insertions (“I”) or deletions (“D”) of the indicated nucleotides at the indicated positions within the repressor region NGS amplicon (see supplemtal text below). Globin transcript abundance by RNA-seq (as %of all globin transcripts) is indicated.

| NGS genotype | NGS allele 1 | NGS Allele 2 | %HBG1  +HBG2 | %HBB | %HBD | %HBA |
| --- | --- | --- | --- | --- | --- | --- |
| PRR (-/-) | D178:1961 |  | 7.88% | 45.20% | 1.67% | 44.51% |
| PRR (+/-) | D184:1979,189(TC->AA) | D1962 (T) |  |  |  |  |
| PRR (-/-) | D172:1962 | D181:1954, 1954ATTCCAC->CTCCAGATC | 1.91% | 48.04% | 1.21% | 48.08% |
| PRR (+/-) | D178:1963 | D1963, D180-183 |  |  |  |  |
| PRR (+/-) | D180:1962 | D1962 (T) |  |  |  |  |
| PRR (-/-) | D178:1961 |  | 14.79% | 38.42% | 1.31% | 44.33% |
| PRR (-/-) | D178:1961 | D178:1961 I(1961):TGT | 0.54% | 49.80% | 1.44% | 47.75% |
| PRR (-/-) | D178:1961 |  | 1.53% | 46.73% | 1.88% | 49.23% |
| Region 4 (+/-) | D661:1034, 1034CACCA:ctctg | I665:G | 2.56% | 45.88% | 2.27% | 48.45% |
| Region 4 (+/-) | D662:1089 | D663:669 | 3.34% | 47.24% | 2.39% | 46.11% |
| Region 4 (+/-) | D661:1034, 1034CACCA:ctctg | D664-667 | 3.95% | 41.81% | 1.45% | 52.27% |
| Region 4 (+/-) | D666:1039 | D1024:1061 |  |  |  |  |
| Region 4 (+/-) | D666:1038 | D666:674 |  |  |  |  |
| Region 4 (+/-) | D667:1038 | Unedited |  |  |  |  |
| Region 4 (-/-) | D666:682,691:1543 | D656:986 | 7.33% | 36.29% | 0.88% | 54.91% |
| Region 4 (-/-) | D661:1034 |  | 13.60% | 30.42% | 1.02% | 54.51% |
| Region 9 (+/-) | D1517:1850 | D1844 |  |  |  |  |
| Region 9 (+/-) | D1512:1844 | I1841:TC |  |  |  |  |
| Region 9 (+/-) | D1517: S1512:5:TAGA | Unedited |  |  |  |  |
| Region 9 (-/-) | D1517:1849, Mut1512-6:TAGAG |  | 15.03% | 23.38% | 1.45% | 58.75% |
| Region 9 (-/-) | D1512:1844 |  | 2.70% | 42.15% | 2.23% | 52.05% |
| Region 9 (-/-) | D1508:1843 ,I1507:GC, M1844:A | D1508:1843 ,S1844:A | 26.04% | 24.20% | 1.09% | 47.75% |
| Region 9 (-/-) | D1512:1844 |  | 0.20% | 41.31% | 1.71% | 56.26% |
| Region 9 (+/-) | D1512:1844 | D1845:1846 | 4.08% | 46.26% | 2.09% | 46.96% |
| Region 9 (+/-) | D1511:1845 | D1845:1849 | 7.34% | 43.54% | 2.67% | 45.57% |
| Region 9 (+/-) | D1512:1844 | M1530:T-G,D1841:2 | 8.72% | 30.19% | 0.45% | 60.38% |
| wild-type | Unedited |  | 11.97% | 33.45% | 1.28% | 52.34% |
| wild-type | Unedited |  | 4.78% | 39.05% | 1.72% | 53.71% |
| wild-type | Unedited |  | 5.27% | 42.32% | 2.46% | 49.07% |
| wild-type | Unedited |  | 7.94% | 41.44% | 1.83% | 47.99% |
| wild-type | Unedited |  | 26.46% | 24.10% | 0.99% | 47.49% |
| wild-type | Unedited |  | 1.50% | 43.56% | 1.45% | 52.96% |
| wild-type | Unedited |  | 13.38% | 37.09% | 1.92% | 46.68% |

**Table c. DNA Oligonucleotides used in this study, including gRNA protospacer sequences where indicated.**

| **Oligo Name** | **Oligo type** | **Sequence** |
| --- | --- | --- |
| Corfu 5' RNA1 | FwdVar for IVT with protospacer | GGATCCTAATACGACTCACTATAGtctttggggtggacacatagGTTTTAGAGCTAGAA |
| Corfu 5' RNA2 | FwdVar for IVT with protospacer | GGATCCTAATACGACTCACTATAGgggtgctacatacttcctaaGTTTTAGAGCTAGAA |
| Corfu 5' RNA3 | FwdVar for IVT with protospacer | GGATCCTAATACGACTCACTATAGcccactcaagagatatggtgGTTTTAGAGCTAGAA |
| Corfu 5' RNA4 | FwdVar for IVT with protospacer | GGATCCTAATACGACTCACTATAGggttttgggattaatgaaacGTTTTAGAGCTAGAA |
| Corfu 3' RNA1 | FwdVar for IVT with protospacer | GGATCCTAATACGACTCACTATAGgttaacagagaaataaagacGTTTTAGAGCTAGAA |
| Corfu 3' RNA2 | FwdVar for IVT with protospacer | GGATCCTAATACGACTCACTATAGagcacattgcccaagagctgGTTTTAGAGCTAGAA |
| Corfu 3' RNA3 | FwdVar for IVT with protospacer | GGATCCTAATACGACTCACTATAGgttaacagagaaataaagacGTTTTAGAGCTAGAA |
| Corfu 3' RNA4 | FwdVar for IVT with protospacer | GGATCCTAATACGACTCACTATAGcttagacaaaactgatccccGTTTTAGAGCTAGAA |
| Repressor 5' RNA1 | FwdVar for IVT with protospacer | GGATCCTAATACGACTCACTATAGaccaggtcaagaatacagaaGTTTTAGAGCTAGAA |
| Repressor 5' RNA2 | FwdVar for IVT with protospacer | GGATCCTAATACGACTCACTATAGagctcagaaagaatacaacGTTTTAGAGCTAGAA |
| Repressor 5' RNA3 | FwdVar for IVT with protospacer | GGATCCTAATACGACTCACTATAGcagtcgtgaacacaagaataGTTTTAGAGCTAGAA |
| Repressor 5' RNA4 | FwdVar for IVT with protospacer | GGATCCTAATACGACTCACTATAGtgtacttagatatgtggatcGTTTTAGAGCTAGAA |
| Repressor 3' RNA1 | FwdVar for IVT with protospacer | GGATCCTAATACGACTCACTATAGagtgttttaggctaatatagGTTTTAGAGCTAGAA |
| Repressor 3' RNA2 | FwdVar for IVT with protospacer | GGATCCTAATACGACTCACTATAGtctgcaaaaatgaaactaggGTTTTAGAGCTAGAA |
| Repressor 3' RNA3 | FwdVar for IVT with protospacer | GGATCCTAATACGACTCACTATAGctgctgaaagagatgcggtgGTTTTAGAGCTAGAA |
| Repressor 3' RNA4 | FwdVar for IVT with protospacer | GGATCCTAATACGACTCACTATAGaactgctgaaagagatgcggGTTTTAGAGCTAGAA |
| Corfu 5' PCR Primer | genomic DNA PCR amplification | gaaacagagagtcagagatgacag |
| Corfu 3' PCR Primer | genomic DNA PCR amplification | tagagcctcaggaaccttcttac |
| PRR 5' PCR Primer | genomic DNA PCR amplification | atgttggtgttcattaaaggtcc |
| PRR 3' Primer | genomic DNA PCR amplification | cttctgagaaactgagccaac |
| Region1 3' RNA | FwdVar for IVT with protospacer | GGATCCTAATACGACTCACTATAGgaaaatgggagacaaatagcGTTTTAGAGCTAGAA |
| Sub-region 1 "stapler" 1S | "Stapler" ssODN | tgttccattctgtattcttgacctggttgtattctttctgagctccagatatttgtctcccattttctctacttctaaaatacatttcttcactaagtga |
| Region2 5' RNA | FwdVar for IVT with protospacer | GGATCCTAATACGACTCACTATAGagctgggcttctgttgcagtGTTTTAGAGCTAGAA |
| Region2 3' RNA | FwdVar for IVT with protospacer | GGATCCTAATACGACTCACTATAGcaaatgcattttacagcattGTTTTAGAGCTAGAA |
| Sub-region 2 "stapler" 2S | "Stapler" ssODN | catggtccaagtctgataatagaaatggcattgtcactttcttccctactgctgtaaaatgcatttgtatcgctacgtgttaagcagtagttgattcttt |
| Region3 5' RNA | FwdVar for IVT with protospacer | GGATCCTAATACGACTCACTATAGcaagttatttttctgtaaccGTTTTAGAGCTAGAA |
| Region3 3' RNA | FwdVar for IVT with protospacer | GGATCCTAATACGACTCACTATAGagcatttctatacatgtcttGTTTTAGAGCTAGAA |
| Sub-region 3 "stapler" 3S | "Stapler" ssODN | atgccaccacctttcttgaattattcaatatctttcgttggcttccaggtctttggatacacacatgcatgtgtttctgaatatctaaaaatgtaattgc |
| Region4 5' RNA | FwdVar for IVT with protospacer | GGATCCTAATACGACTCACTATAGggttgattaaaagtaaccagGTTTTAGAGCTAGAA |
| Region4 3' RNA | FwdVar for IVT with protospacer | GGATCCTAATACGACTCACTATAGactgacagttcaaattttggGTTTTAGAGCTAGAA |
| Sub-region 4 "stapler" 4S | "Stapler" ssODN | atccatctttctatatacaaataaagtcatatagtttgaactcacctctgaaatttgaactgtcagtcttatctcttctcttgtctcttttttcctcttt |
| Region5 5' RNA | FwdVar for IVT with protospacer | GGATCCTAATACGACTCACTATAGagcatttctatacatgtcttGTTTTAGAGCTAGAA |
| Region5 3' RNA | FwdVar for IVT with protospacer | GGATCCTAATACGACTCACTATAGtggagaggagaggagaaaaaGTTTTAGAGCTAGAA |
| Sub-region 5 "stapler" 5S | "Stapler" ssODN | cggtttgaggtttttatgattattgctgtcataagcatttctatacatgtttctcctctcctctccattatttatttttccttcttctcctccatccctt |
| Region6 5' RNA | FwdVar for IVT with protospacer | GGATCCTAATACGACTCACTATAGactgacagttcaaattttggGTTTTAGAGCTAGAA |
| Region6 3' RNA | FwdVar for IVT with protospacer | GGATCCTAATACGACTCACTATAGagaaaagagtgaaaaactacGTTTTAGAGCTAGAA |
| Sub-region 6 "stapler" 6S | "Stapler" ssODN | agtgtcaccaacagagtttgagtttctattgatccatatcaccaccaaaatactgggatcttcattcctgggtctaattccacatttttttttaagaaca |
| Region7 5' RNA | FwdVar for IVT with protospacer | GGATCCTAATACGACTCACTATAGagaggagaggagaaaaaaggGTTTTAGAGCTAGAA |
| Region7 3' RNA | FwdVar for IVT with protospacer | GGATCCTAATACGACTCACTATAGgaacttcgttaaatttaatcGTTTTAGAGCTAGAA |
| Sub-region 7 "stapler" 7S | "Stapler" ssODN | ttccctttctctttctcttccctatcccttctcctctcctctcccctcctatctggctgaatatggtaaatctacttttcactttgcattctttctttag |
| Region8 5' RNA | FwdVar for IVT with protospacer | GGATCCTAATACGACTCACTATAGgaattcaaataatgccacaaGTTTTAGAGCTAGAA |
| Region8 3' RNA | FwdVar for IVT with protospacer | GGATCCTAATACGACTCACTATAGccagaacctatttcaataacGTTTTAGAGCTAGAA |
| Sub-region 8 "stapler" 8S | "Stapler" ssODN | tcaatccttccttttggatatgctcatgggtgtgtatttgtctgccattgaactggtaaataaacactattcattttttaaatattcttttaatggatat |
| Region9 5' RNA | FwdVar for IVT with protospacer | GGATCCTAATACGACTCACTATAGaaatttaatctggctgaataGTTTTAGAGCTAGAA |
| Region9 3' RNA | FwdVar for IVT with protospacer | GGATCCTAATACGACTCACTATAGttggagacaaaaatctctagGTTTTAGAGCTAGAA |
| Sub-region 9 "stapler" 9S | "Stapler" ssODN | gttctgtactagcatattcccaggaacttcgttaaatttaatctggctgatagaggcaaagaagaactttatattgagtcaacttgttaaaacatctgct |
| Sub-region 4 guide -5 (5' RNA1) | FwdVar for IVT with protospacer | GGATCCTAATACGACTCACTATAGatatagtttgaactcacctcGTTTTAGAGCTAGAA |
| Sub-region 4 guide 21 (5' RNA2) | FwdVar for IVT with protospacer | GGATCCTAATACGACTCACTATAGcaaatgcattttacagcattGTTTTAGAGCTAGAA |
| Sub-region 4 guide 111 (5' RNA3) | FwdVar for IVT with protospacer | GGATCCTAATACGACTCACTATAGacagtagaattgataaattaGTTTTAGAGCTAGAA |
| Sub-region 4 guide 125 (5' RNA4) | FwdVar for IVT with protospacer | GGATCCTAATACGACTCACTATAGtaatttatcaattctactgtGTTTTAGAGCTAGAA |
| Sub-region 4 guide 140 | FwdVar for IVT with protospacer | GGATCCTAATACGACTCACTATAGactgttggtaagcatttaagGTTTTAGAGCTAGAA |
| Sub-region 4 guide 147 | FwdVar for IVT with protospacer | GGATCCTAATACGACTCACTATAGgtaagcatttaagtggctacGTTTTAGAGCTAGAA |
| Sub-region 4 guide 154 | FwdVar for IVT with protospacer | GGATCCTAATACGACTCACTATAGtttaagtggctaccggtttgGTTTTAGAGCTAGAA |
| Sub-region 4 guide 155 | FwdVar for IVT with protospacer | GGATCCTAATACGACTCACTATAGtaatcataaaaacctcaaacGTTTTAGAGCTAGAA |
| Sub-region 4 guide 200 | FwdVar for IVT with protospacer | GGATCCTAATACGACTCACTATAGagcatttctatacatgtcttGTTTTAGAGCTAGAA |
| Sub-region 4 guide 248 | FwdVar for IVT with protospacer | GGATCCTAATACGACTCACTATAGtatctaaaaatgtaattgctGTTTTAGAGCTAGAA |
| Sub-region 4 guide 279 | FwdVar for IVT with protospacer | GGATCCTAATACGACTCACTATAGttaatagtatccacaaatgcGTTTTAGAGCTAGAA |
| Sub-region 4 guide 280 | FwdVar for IVT with protospacer | GGATCCTAATACGACTCACTATAGtatcaagcatccagcatttgGTTTTAGAGCTAGAA |
| Sub-region 4 guide 293 | FwdVar for IVT with protospacer | GGATCCTAATACGACTCACTATAGgcatttgtggatactattaaGTTTTAGAGCTAGAA |
| Sub-region 4 guide 304 | FwdVar for IVT with protospacer | GGATCCTAATACGACTCACTATAGtactattaaaggttttccaaGTTTTAGAGCTAGAA |
| Sub-region 4 guide 305 | FwdVar for IVT with protospacer | GGATCCTAATACGACTCACTATAGactattaaaggttttccaaaGTTTTAGAGCTAGAA |
| Sub-region 4 guide 306 (3' RNA1) | FwdVar for IVT with protospacer | GGATCCTAATACGACTCACTATAGctattaaaggttttccaaagGTTTTAGAGCTAGAA |
| Sub-region 4 guide 309 (3' RNA2) | FwdVar for IVT with protospacer | GGATCCTAATACGACTCACTATAGgtacaatagtataaccccttGTTTTAGAGCTAGAA |
| Sub-region 4 guide 339 (3' RNA3) | FwdVar for IVT with protospacer | GGATCCTAATACGACTCACTATAGaatagaaactcaaactctgtGTTTTAGAGCTAGAA |
| Sub-region 4 guide 365 | FwdVar for IVT with protospacer | GGATCCTAATACGACTCACTATAGttcaaattttggtggtgataGTTTTAGAGCTAGAA |
| Sub-region 4 guide 376 (3' RNA4) | FwdVar for IVT with protospacer | GGATCCTAATACGACTCACTATAGaagactgacagttcaaatttGTTTTAGAGCTAGAA |
| Region 9 5' RNA1 | FwdVar for IVT with protospacer | GGATCCTAATACGACTCACTATAGgaacttcgttaaatttaatcGTTTTAGAGCTAGAA |
| Region 9 5' RNA2 | FwdVar for IVT with protospacer | GGATCCTAATACGACTCACTATAGattttgaatgtttaaaattaGTTTTAGAGCTAGAA |
| Region 9 5' RNA3 | FwdVar for IVT with protospacer | GGATCCTAATACGACTCACTATAGttctggaaacatgaattttaGTTTTAGAGCTAGAA |
| Region 9 5' RNA4 | FwdVar for IVT with protospacer | GGATCCTAATACGACTCACTATAGccagttattgaaataggttcGTTTTAGAGCTAGAA |
| Region 9 3' RNA1 | FwdVar for IVT with protospacer | GGATCCTAATACGACTCACTATAGgtctccaagggaattttgagGTTTTAGAGCTAGAA |
| Region 9 3' RNA2 | FwdVar for IVT with protospacer | GGATCCTAATACGACTCACTATAGccaacctctcaaaattccctGTTTTAGAGCTAGAA |
| Region 9 3' RNA3 | FwdVar for IVT with protospacer | GGATCCTAATACGACTCACTATAGctagagatttttgtctccaaGTTTTAGAGCTAGAA |
| Region 9 3' RNA4 | FwdVar for IVT with protospacer | GGATCCTAATACGACTCACTATAGtggagacaaaaatctctagaGTTTTAGAGCTAGAA |
| RevLong | gRNA RevLong for IVT | AAAAAAGCACCGACTCGGTGCCACTTTTTCAAGTTGATAACGGACTAGCCTTATTTTAACTTGCTATTTCTAGCTCTAAAAC |
| T7FwdAmp | Amplification primer for IVT template | GGATCCTAATACGACTCACTATAG |
| T7RevAmp | Amplification primer for IVT template | AAAAAAGCACCGACTCGG |

Supplemental Information

**I. Repressor region PCR amplicon, used as a reference for NGS analysis of clonal cell lines and HSPC colonies.**

AACATAATTCCTCCCTCACAAACACATTCTAAGATTTTAAGGAGATATTGATGAAGTACATCATCTGTCATTTTTAACAGGTAGTGGTAGTGATTCACACAGCACATTATGATCTGTTCTTGTATGTTCTGTTCCATTCTGTATTCTTGACCTGGTTGTATTCTTTCTGAGCTCCAGATCCACATATCTAAGTACATCTTTTTGCATTTTACAAGAGTGCATACAATACAATGTATCCAAGAcTGTATTTCTGATTTTATCGTACCACTAAACTCACAAATGTGGCCCTATTCTTGTGTTCACGACTGACATCACCGTCATGGTCCAAGTCTGATAATAGAAATGGCATTGTCACTTTCTTCCCTACTGCAACAGAAGCCCAGCTATTTGTCTCCCATTTTCTCTACTTCTAAAATACATTTCTTCACTAAGTGAGAATAATCTTTTAAAGACACAAATCAAACCATGCCACCACCTTTCTTGAATTATTCAATATCTTTCGTTGGCTTCCAGGTTACAGAAAAATAACTTGTAACAAAGTTTAAAGGTCATTCATGGCTCCTCTCTACCCTATTTTATAACATTTCCCCTTGTGATCAGAATCTCAGGCACATCATCCATCTTTCTATATACAAATAAAGTCATATAGTTTGAACTCACCTCTGGTTACTTTTAATCAACCAAATGCTGTAAAATGCATTTGTATCGCTACGTGTTAAGCAGTAGTTGATTCTTTTCATTTCTGTGTAATATTCTATTCTTTGACTATACCGTAATTTATCAATTCTACTGTTGGTAAGCATTTAAGTGGCTACCGGTTTGAGGTTTTTATGATTATTGCTGTCATAAGCATTTCTATACATGTCTTTGGATACACACATGCATGTGTTTCTGAATATCTAAAAATGTAATTGCTAGGTAATAGACTTATCAAGCATCCAGCATTTGTGGATACTATTAAAGGTTTTCCAAAGGGGTTATACTATTGTACAGTGTCACCAACAGAGTTTGAGTTTCTATTGATCCATATCACCACCAAAATTTGAACTGTCAGTCTTATCTCTTCTCTTGTCTCTTTTTTCCTCTTTTTTTTCCTTCCCTTCCCCTCTCTTCGTTTCTTTTCTCTCCTCTTCTCTTCTTTCCTCTCTTCCCTTCCCTTTCTCTTTCTCTTCCCTATCCCTTCTCCTCTCCTCTCCCCTCCTTTTTTCTCCTCTCCTCTCCATTATTTATTTTTCCTTCTTCTCCTCCATCCCTTCCATCCTCTCTCTTCCCCTCTTCCTTCCTTCCTTTCTCCATTTCTTCCTCCTCTTTCCTTCAATCCTTCCTTTTGGATATGCTCATGGGTGTGTATTTGTCTGCCATTGTGGCATTATTTGAATTCAGAAAAGAGTGAAAAACTACTGGGATCTTCATTCCTGGGTCTAATTCCACATTTTTTTTTAAGAACACATCTGTAAAAATGTTCTGTACTAGCATATTCCCAGGAACTTCGTTAAATTTAATCTGGCTGAATATGGTAAATCTACTTTTCACTTTGCATTCTTTCTTTAGTCATACCATAATTTTAAACATTCAAAATATTTGTATATAATATTTGATTTTATCTGTCATTAAAATGTTAACCTTAAAATTCATGTTTCCAGAACCTATTTCAATAACTGGTAAATAAACACTATTCATTTTTTAAATATTCTTTTAATGGATATTTATTTCAATATAATAAAAAATTAGAGTTTTATTATAGGAAGAATTTACCAAAAGAAGGAGGAAGCAAGCAAGTTTAAACTGCAGCAATAGATTTGTCCATTCCAACCTCTCAAAATTCCCTTGGAGACAAAAATCTCTAGAGGCAAAGAAGAACTTTATATTGAGTCAACTTGTTAAAACATCTGCTTTTAGATAAGTTTTCTTAGTATAAAGTGACAGAAACAAATAAGTTAAACTCTAAGATACATTCCACTATATTAGCCTAAAACACTTCTGCAAAAATGAAACTAGGAGGATATTTTTAGAAACAACTGCTGAAAGAGATGCGGTGGGGAGATATGTAGAGGAGAACAGGGTTTCTGAGTCAAGACACACATGACAGAACAGCCAATCTCAGGGCAAGTTAAGGGAATAGTGGAATGAAGGTTCATTTTTCATTCTCACAAACTAATGAAACCCTGCTTATCTTAAACCAACCTGCTCACTGGAGCAGGGAGGACAGGACCAGCATAAAAGGCAGGGCAGAGTCGACTGTTGCTTACACTTTCTTCTGACATAACAGTGTTCACTAGCAACCTCAAACAGACACCAT

Key

Sub-region 4

Sub-region 9

HBD 5’ UTR

Putative Repressor Region (PRR)
